# Supplementary material for: Genome-Wide Analysis of Stress-Responsive Genes and Alternative Splice Variants in Arabidopsis Roots under Osmotic Stresses
Source: Int J Mol Sci. 2023 Sep 26;24(19):14580. doi: 10.3390/ijms241914580 (PMC10573044; doi:10.3390/ijms241914580)
Supplement: Supplementary file 1 [file ijms-24-14580-s001.zip › ijms-2546419-supplementary data-2023-09-04.pdf]

Supplementary data

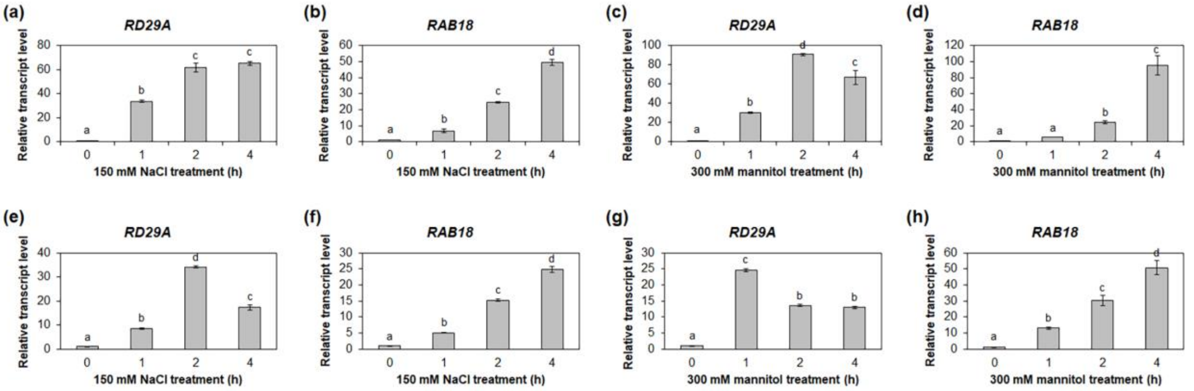

Figure S1. Expression analysis of osmotic stress-responsive marker genes. Quantitative RT-PCR analysis of *RD29A* and *RAB18* in whole seedlings (**a**–**d**) and in roots (**e**–**h**) under 150 mM NaCl or 300 mM mannitol treatment for 0, 1, 2, and 4 h. *GAPc* was used as an internal control. Transcript levels at 0 h were set as 1. Three biological replicates were performed with two technical replicates for each biological replicate. Three independent reactions were performed for each technical replicate. Error bars represent standard deviation (n = 6 reactions). Different letters indicate statistically significant differences ($p < 0.05$).

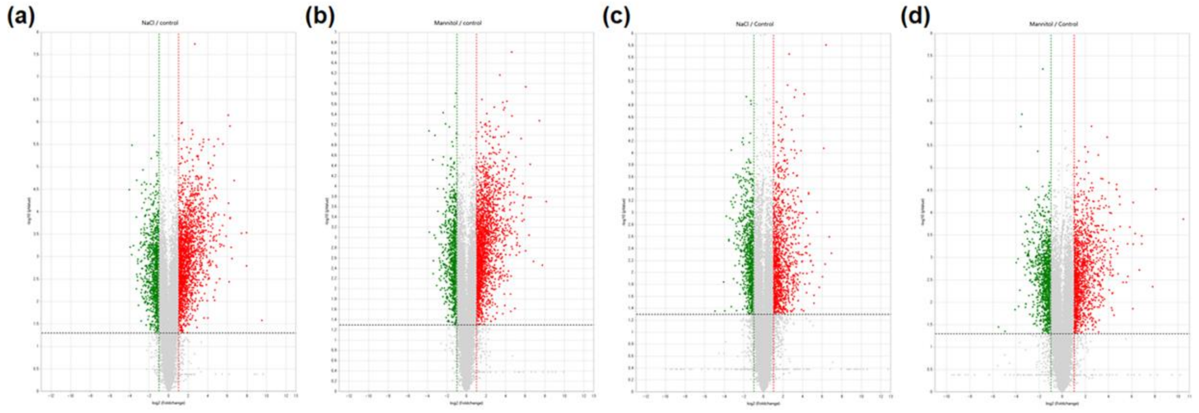

Figure S2. Volcano plot of differentially expressed genes (DEGs) under osmotic stress conditions. (**a**) DEGs in roots under NaCl treatment. (**b**) DEGs in roots under mannitol treatment. (**c**) DEGs in whole seedlings under NaCl treatment. (**d**) DEGs in whole seedlings under mannitol treatment. Red dots and green dots represent upregulated genes and downregulated genes, respectively. Grey dots indicate genes that are not differentially expressed. DEGs were selected by FDR < 0.05 and log2 ratio ≥ 1 or ≤ −1 condition. Volcano plots were visualized using ExDEGA.

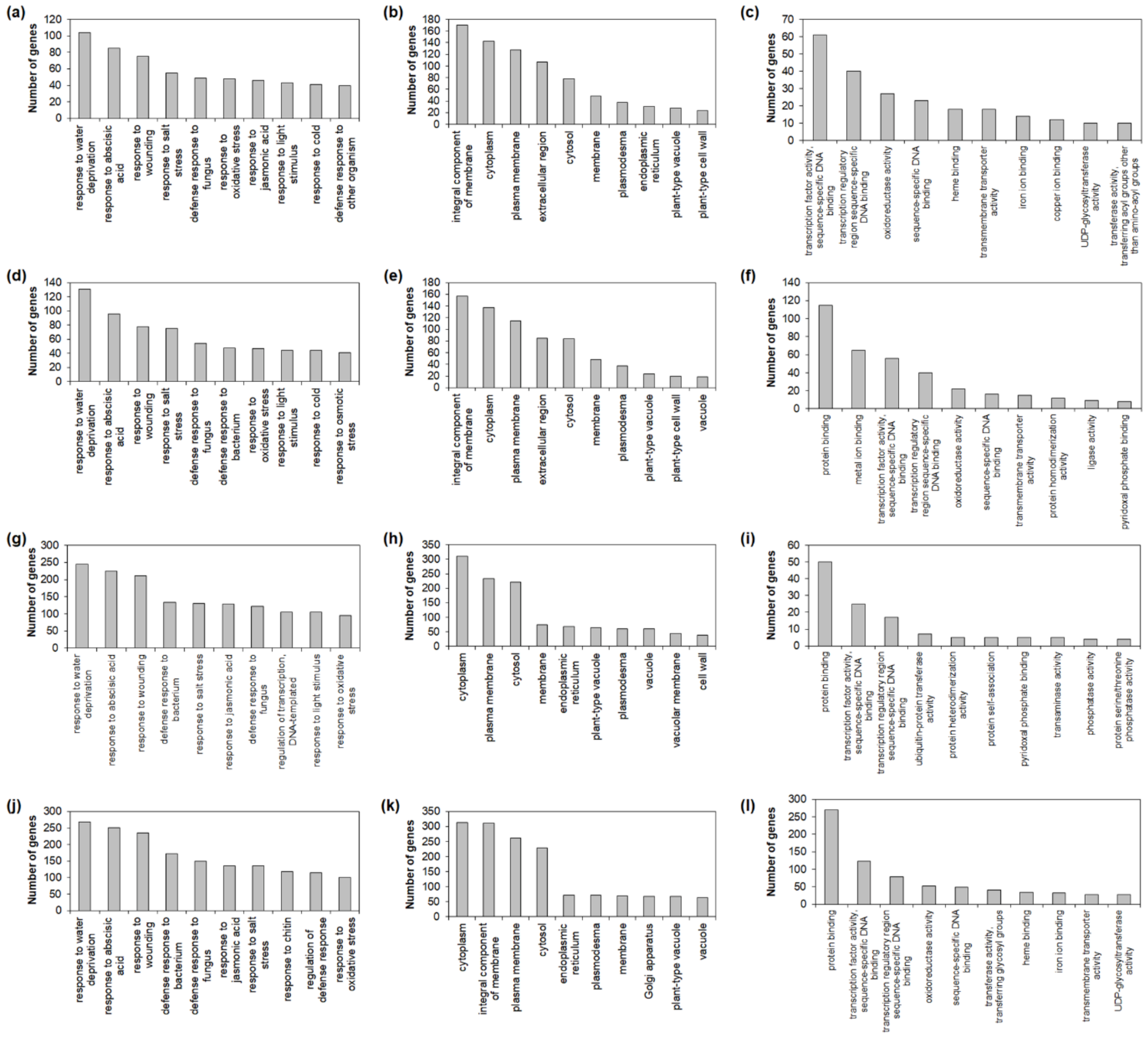

Figure S3. Enriched gene ontology (GO) terms of upregulated genes in roots and in whole seedlings under salt and drought stress conditions. (**a–c**) Enriched Biological Process (BP) (**a**), Molecular Function (MF) (**b**), and Cellular Component (CC) (**c**) of upregulated genes in roots under NaCl treatment. (**d–f**) Enriched BP (**d**), MF (**e**), and CC (**f**) of upregulated genes in roots under mannitol treatment. (**g–i**) Enriched BP (**g**), MF (**h**), and CC (**i**) of upregulated genes in whole seedlings under NaCl treatment. (**j–l**) Enriched BP (**j**), MF (**k**), and CC (**l**) of upregulated genes in whole seedlings under mannitol treatment. GO annotation enrichment was performed using DAVID. Enriched GO terms were selected by $p < 0.05$.

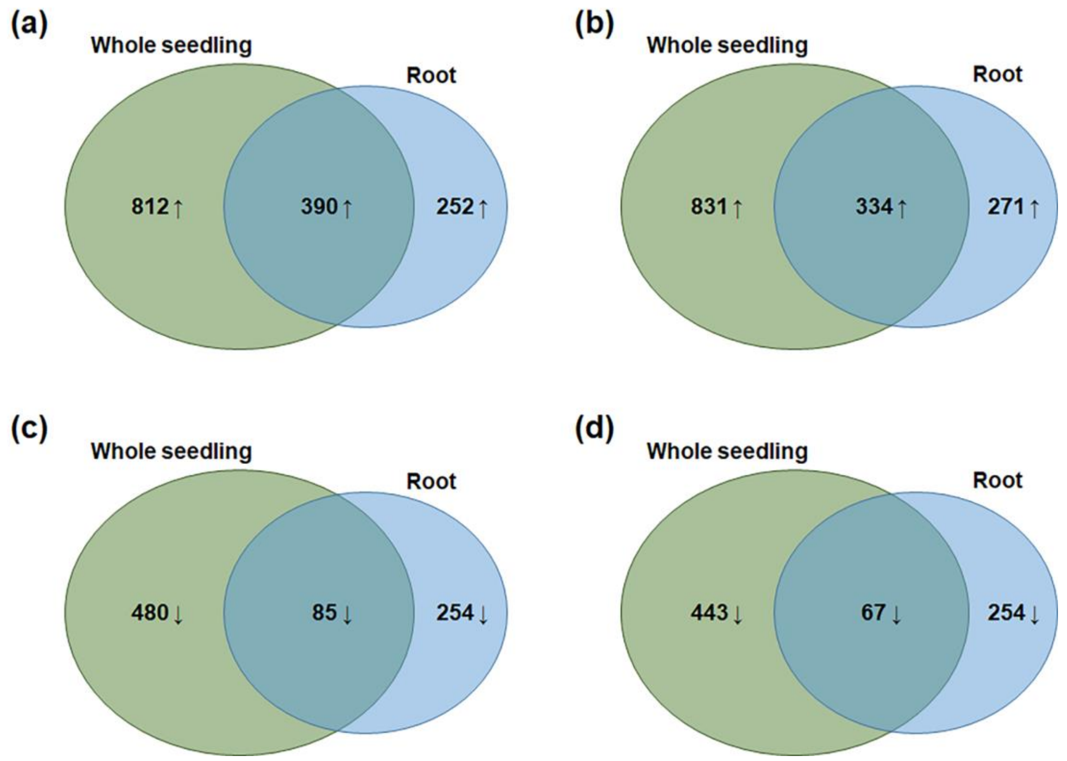

Figure S4. Venn diagrams of DEGs in whole seedlings and in roots. (**a**) Venn diagram of upregulated genes in whole seedlings and in roots under NaCl treatment. (**b**) Venn diagram of upregulated genes in whole seedlings and in roots under mannitol treatment. (**c**) Venn diagram of downregulated genes in whole seedlings and in roots under NaCl treatment. (**d**) Venn diagram of downregulated genes in whole seedlings and in roots under mannitol treatment. DEGs were selected by FDR < 0.05 and log2 ratio ≥ 1 or ≤ -1 condition.

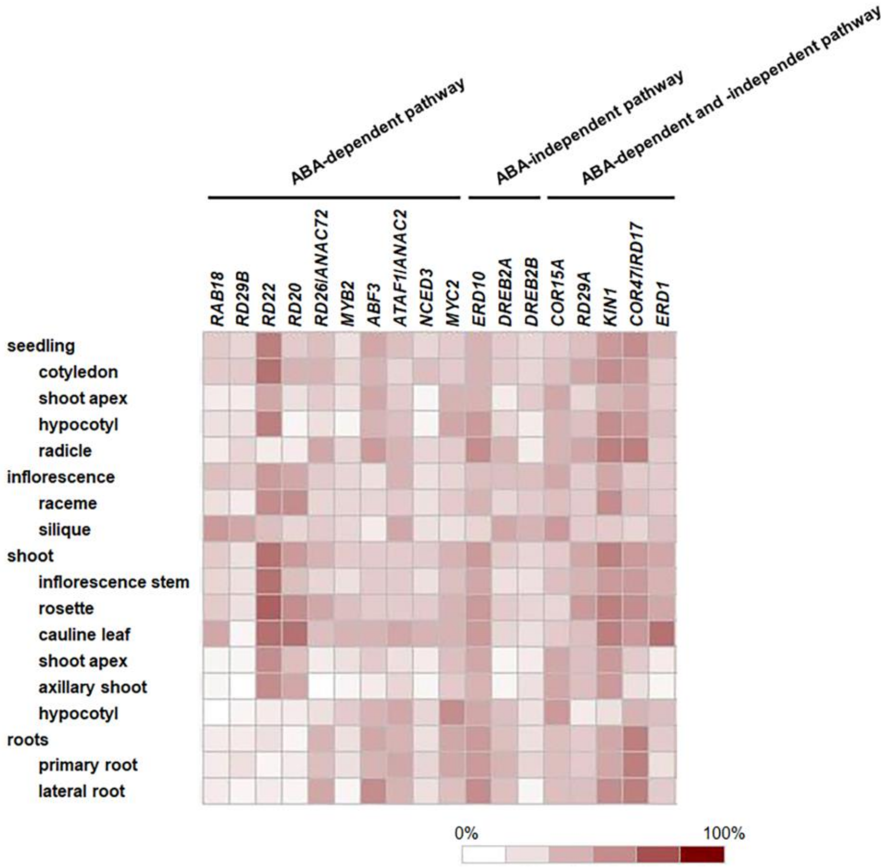

Figure S5. Heatmap analysis of the ABA-dependent and -independent genes in Arabidopsis organs using Genevestigator. Genevestigator analysis was performed by organism as *Arabidopsis thaliana*, platform as Affymetrix Arabidopsis ATH1 Genome Array, and condition search tool as Anatomy.

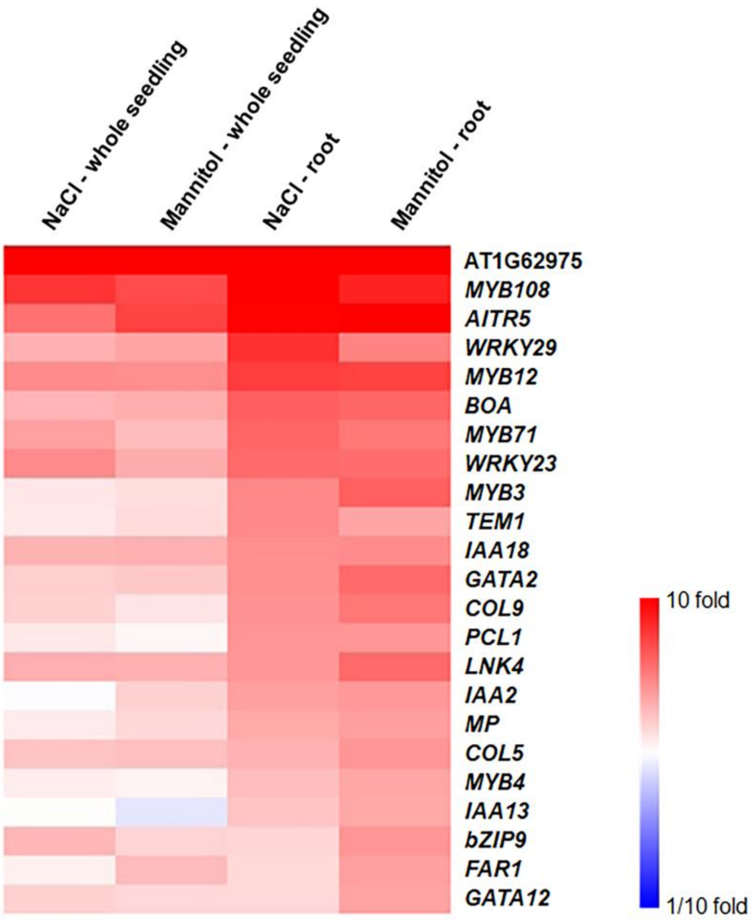

Figure S6. Expression of transcription factor genes upregulated under osmotic stress conditions in roots. Expression of transcription factor genes upregulated in roots were visualized using MeV. MeV was performed by Two-color Array, Load annotation data as Automatically download, and organism as *Arabidopsis thaliana*.

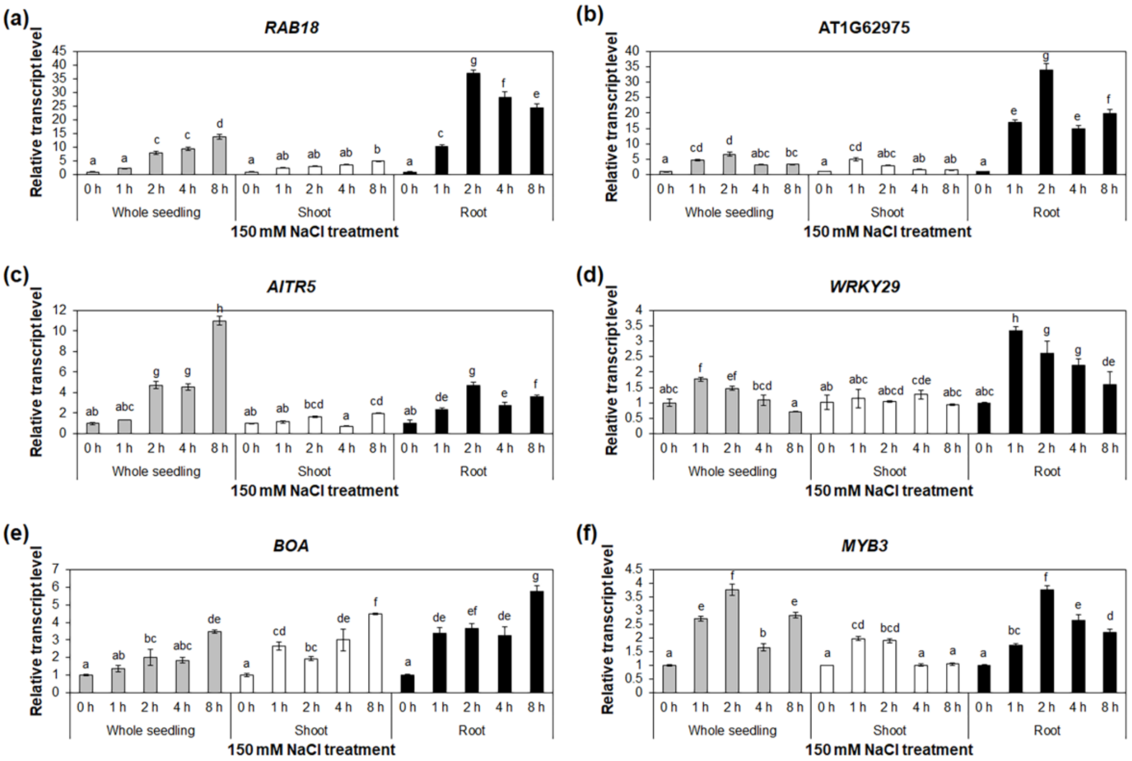

Figure S7. Expression analysis of salt stress-responsive transcription factor gene. Quantitative RT-PCR analysis of *RAB18* (**a**), AT1G62975 (**b**), *AITR5* (**c**), *WRKY29* (**d**), *BOA* (**e**), and *MYB3* (**f**) in whole seedlings, shoots, and roots under 150 mM NaCl treatment for 0, 1, 2, 4, and 8 h. *GAPc* was used as an internal control. Transcript levels at 0 h in whole seedlings, shoots, or roots were set as 1. Three biological replicates were performed with two technical replicate for each biological replicate. Three independent reactions were performed for each technical replicate. Error bars represent standard deviation ($n = 6$ reactions). Different letters indicate statistically significant differences ($p < 0.05$).

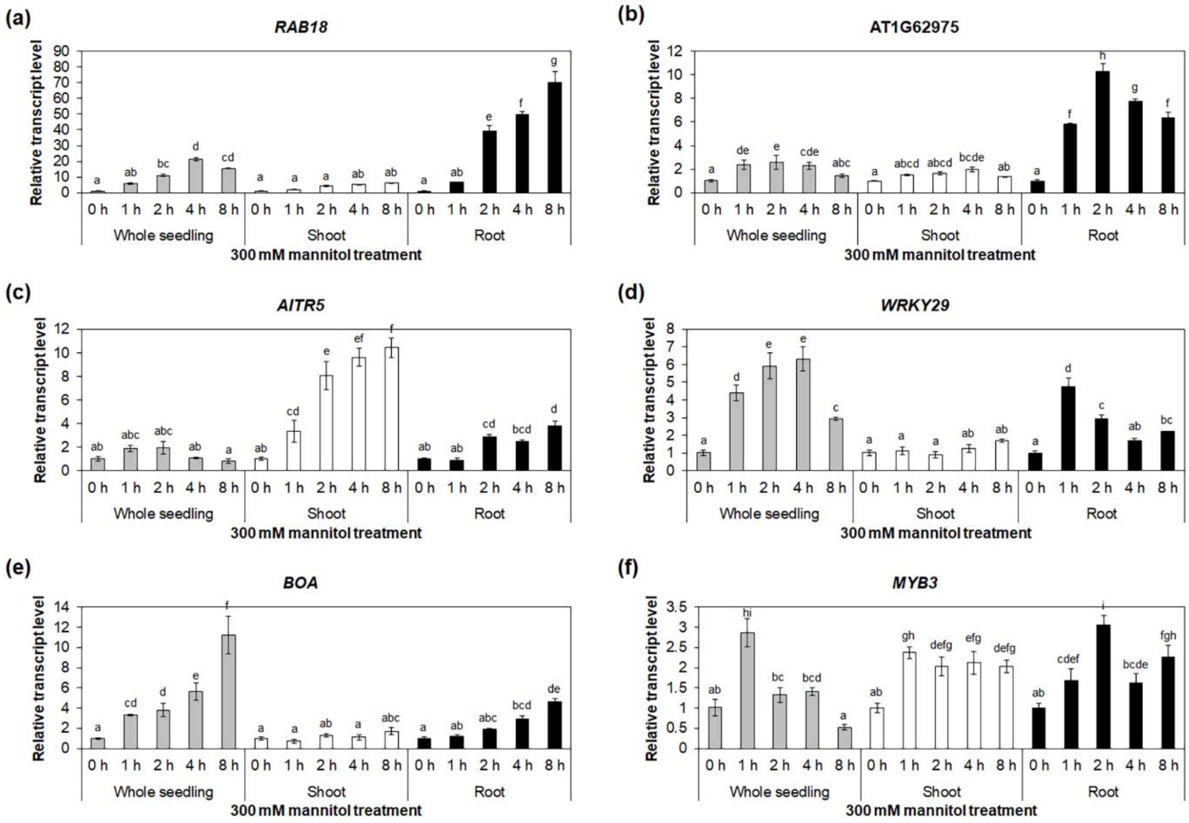

Figure S8. Expression analysis of drought stress-responsive transcription factor gene. Quantitative RT-PCR analysis of *RAB18* (**a**), AT1G62975 (**b**), *AITR5* (**c**), *WRKY29* (**d**), *BOA* (**e**), and *MYB3* (**f**) in whole seedlings, shoots, and roots under 300 mM mannitol treatment for 0, 1, 2, 4, and 8 h. *GAPc* was used as an internal control. Transcript levels at 0 h in whole seedlings, shoots, or roots were set as 1. Two biological replicates were performed with two technical replicate for each biological replicate. Three independent reactions were performed for each technical replicate. Error bars represent standard deviation ($n = 4$ reactions). Different letters indicate statistically significant differences ($p < 0.05$).

(a)

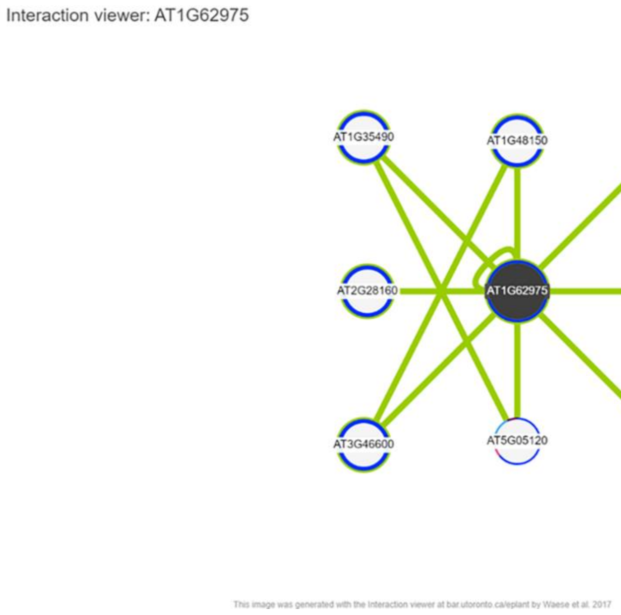

Figure S9. Protein-protein or protein-DNA interactions of osmotic stress-responsive transcription factor genes using BAR analysis. BAR analysis was performed using Interaction viewer in ePlant.

(b)

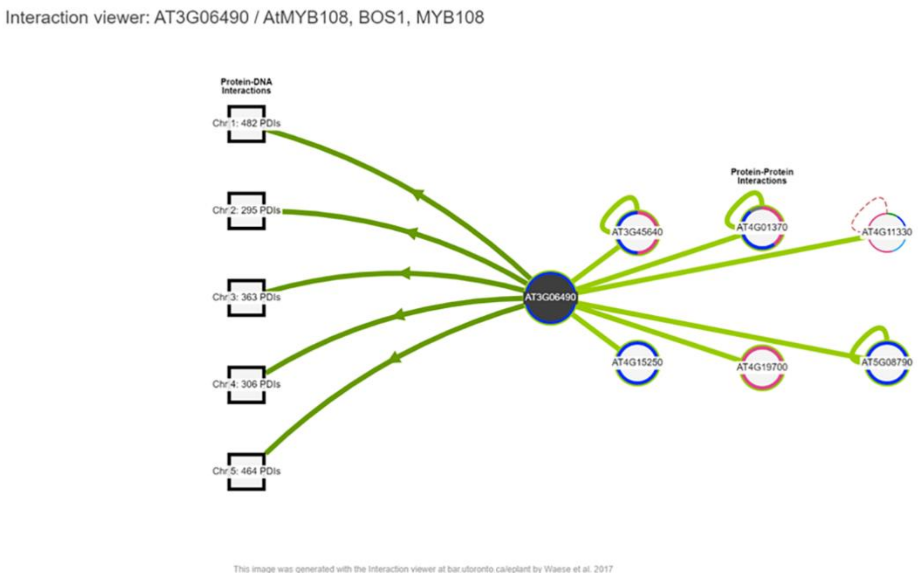

Figure S9. Continued.

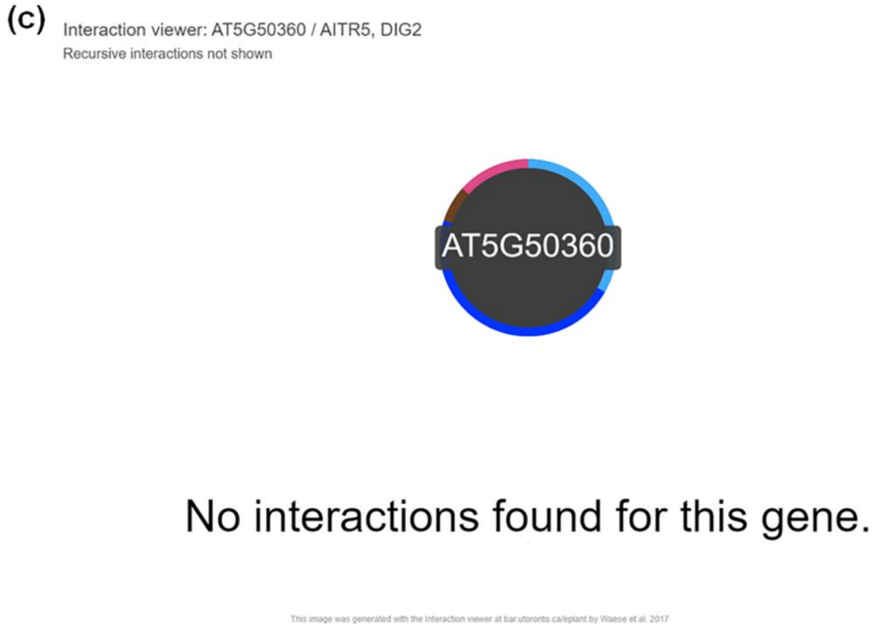

Figure S9. Continued.

(d)

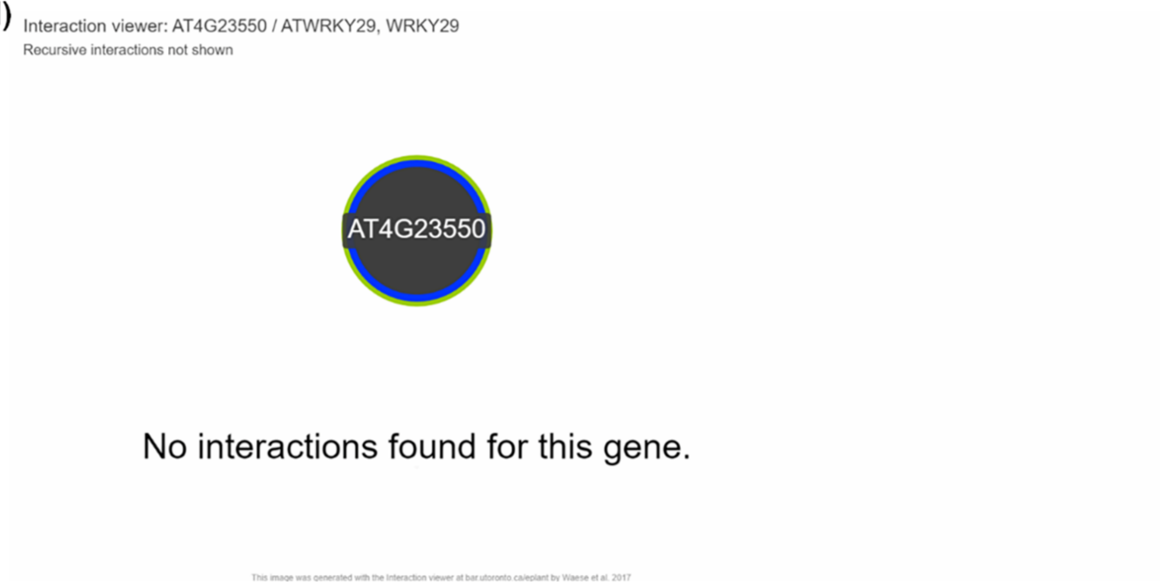

Figure S9. Continued.

(e) Interaction viewer: AT2G47460 / ATMYB12, MYB12, PFG1

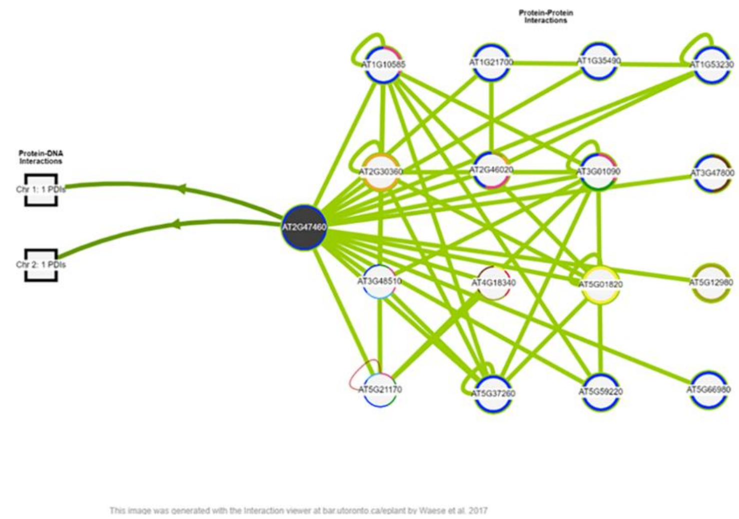

Figure S9. Continued.

(f) Interaction viewer: AT5G59570 / BOA

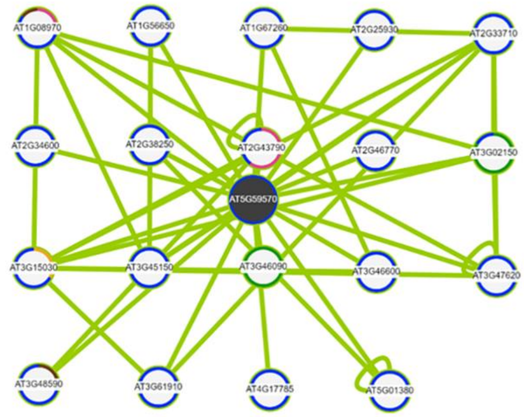

This image was generated with the Interaction viewer at bar.utoronto.ca/eplant by Waese et al. 2017

Figure S9. Continued.

(g) Interaction viewer: AT3G24310 / ATMYB71, MYB305

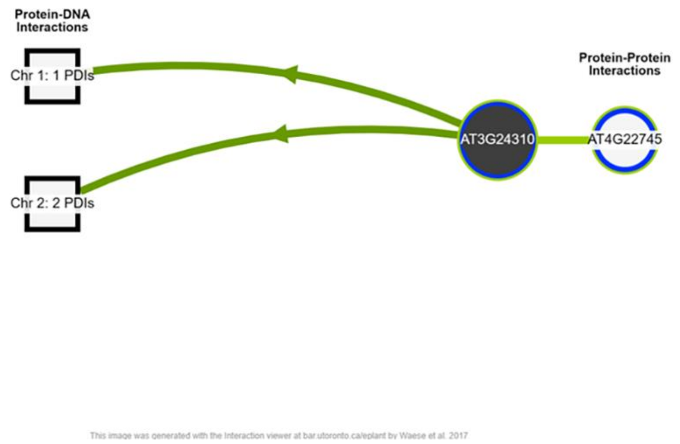

Figure S9. Continued.

(h) Interaction viewer: AT2G47260 / ATWRKY23, WRKY23

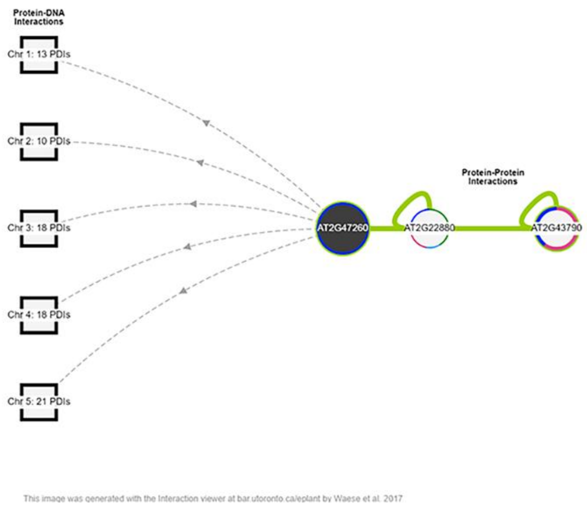

Figure S9. Continued.

(i) Interaction viewer: AT1G22640 / ATMYB3, MYB3

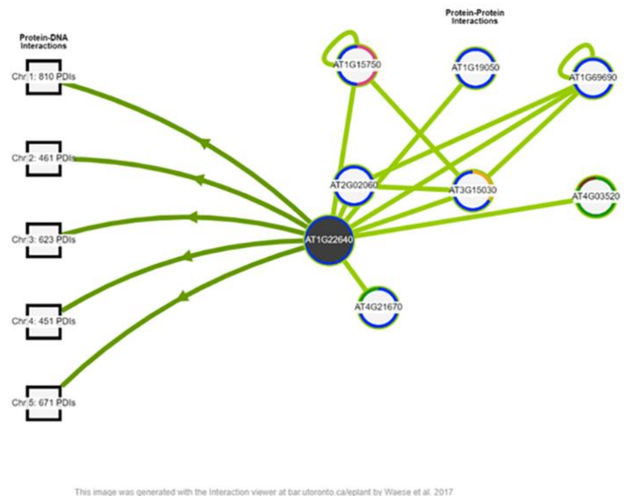

Figure S9. Continued.

(j)

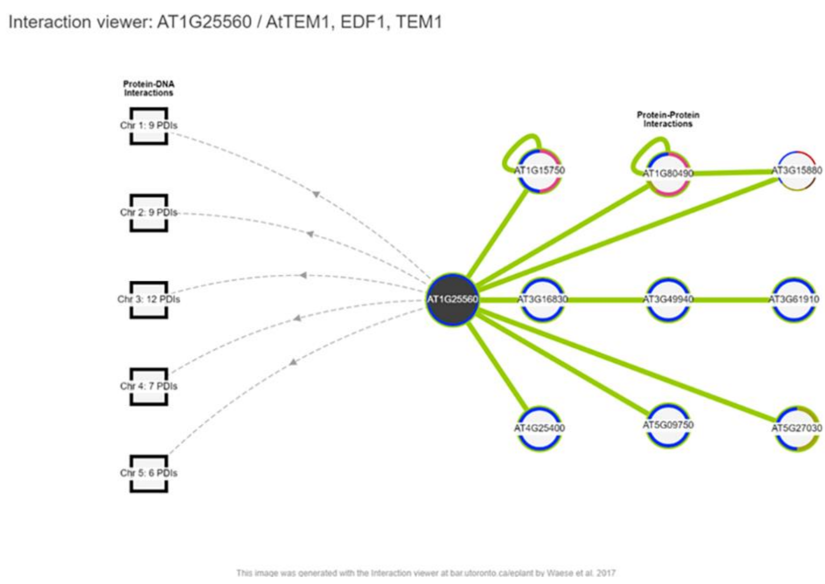

Figure S9. Continued.

(k) Interaction viewer: AT1G51950 / IAA18

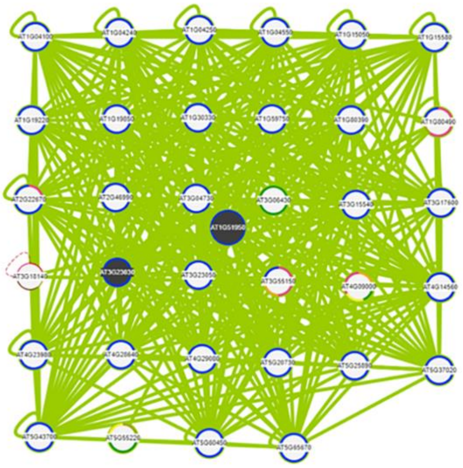

This image was generated with the Interaction viewer at bar.utoronto.ca/eplant by Waese et al. 2017

Figure S9. Continued.

(l)

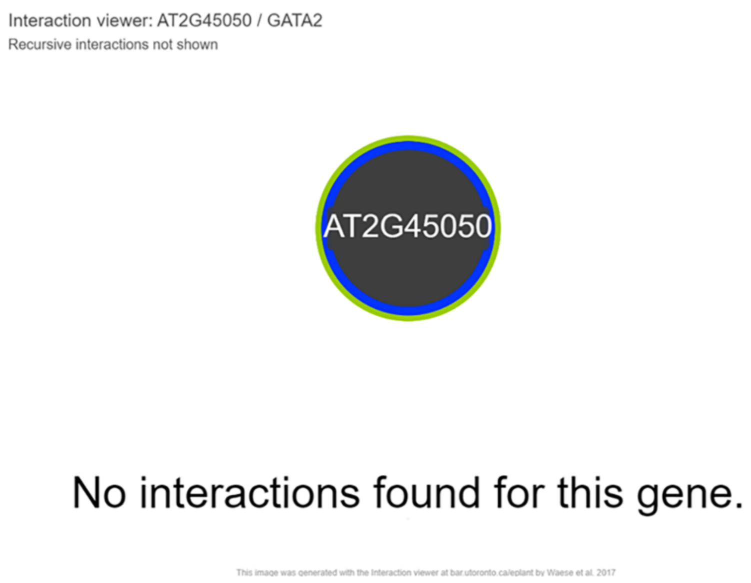

Figure S9. Continued.

(m) Interaction viewer: AT3G07650 / BBX7, COL9

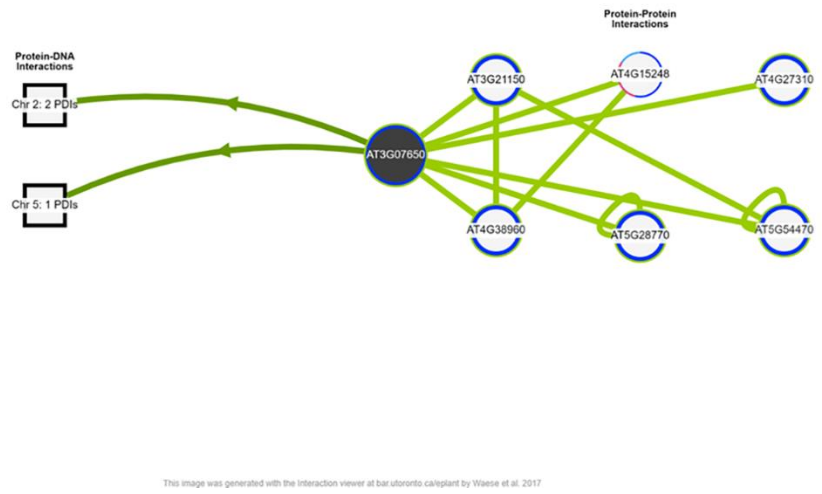

Figure S9. Continued.

(n) Interaction viewer: AT3G46640 / LUX, PCL1

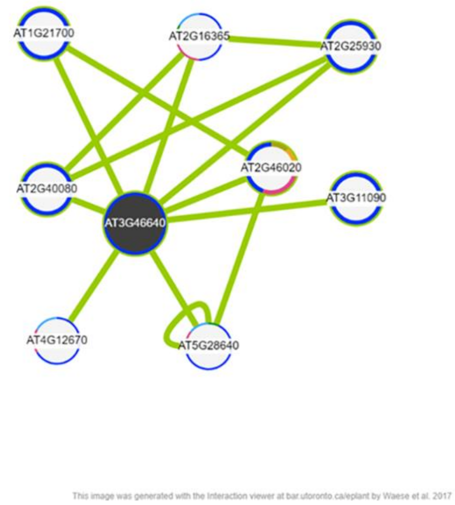

Figure S9. Continued.

(o)

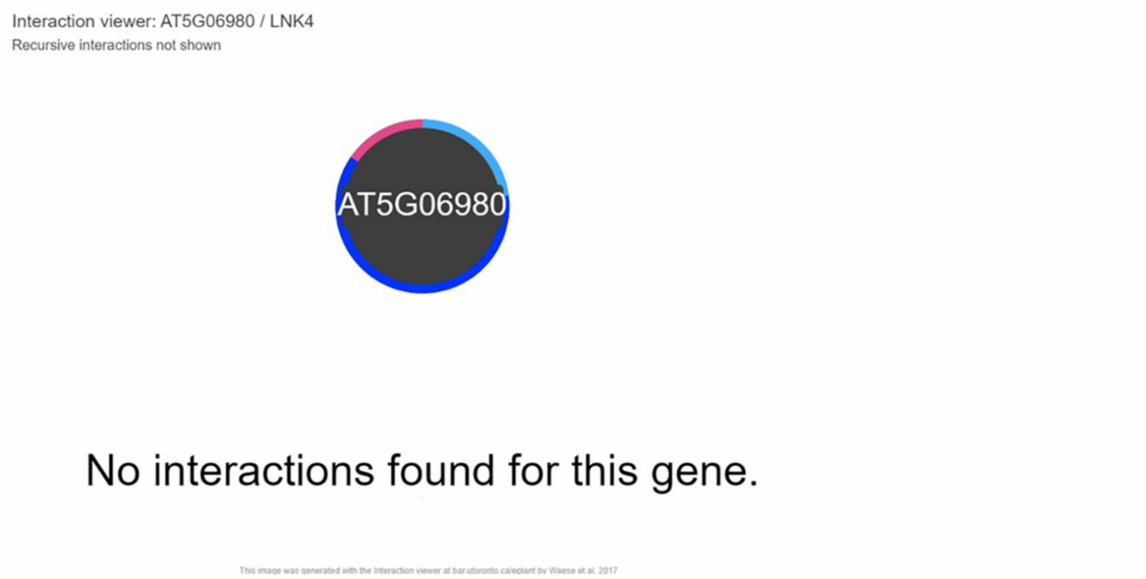

Figure S9. Continued.

(p) Interaction viewer: AT3G23030 / IAA2

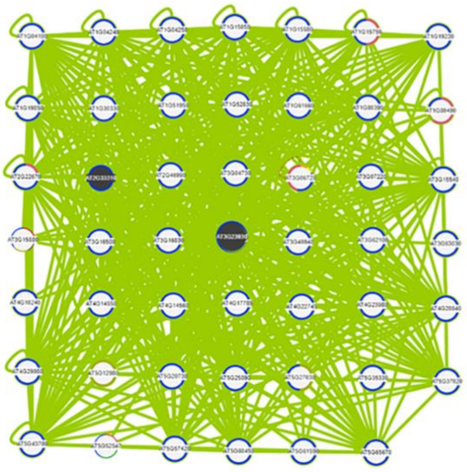

This image was generated with the Interaction viewer at bar.utoronto.ca/eplant by Waese et al. 2017

Figure S9. Continued.

(q) Interaction viewer: AT1G19850 / ARF5, IAA24, MP

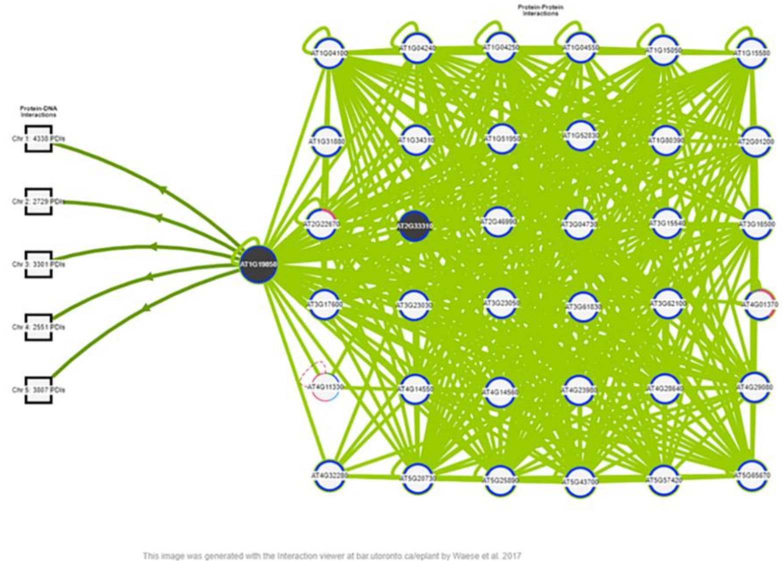

Figure S9. Continued.

(r) Interaction viewer: AT5G57660 / ATCOL5, BBX6, COL5

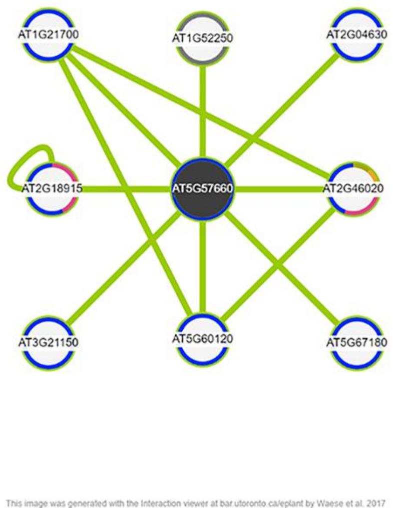

Figure S9. Continued.

(s) Interaction viewer: AT4G38620 / ATMYB4, MYB4

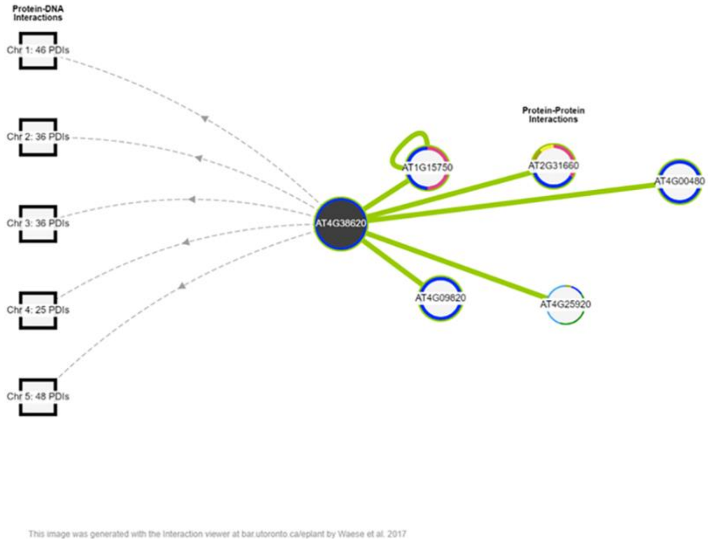

Figure S9. Continued.

(t) Interaction viewer: AT2G33310 / IAA13

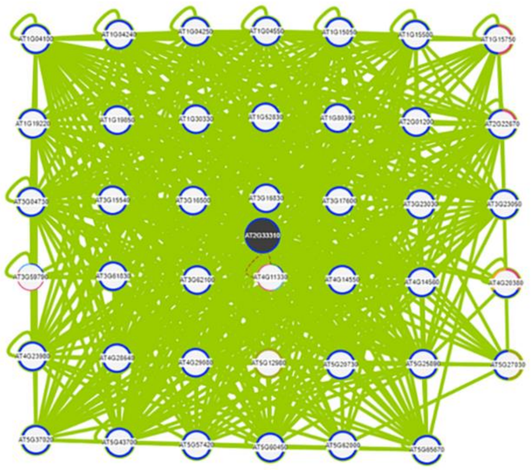

This image was generated with the Interaction viewer at bar.utoronto.ca/eplant by Waese et al. 2017

Figure S9. Continued.

(u) Interaction viewer: AT5G24800 / ATBZIP9, BZIP9, BZO2H2

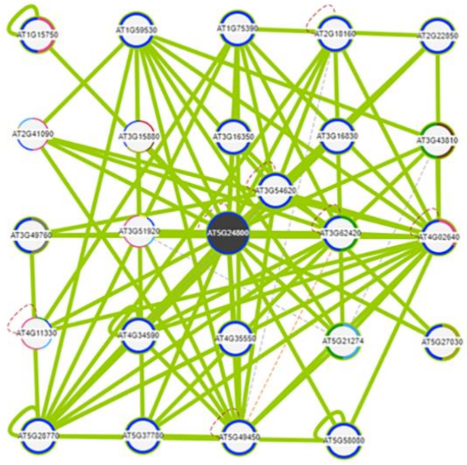

This image was generated with the Interaction viewer at bar.utoronto.ca/eplant by Waese et al. 2017

Figure S9. Continued.

(v) Interaction viewer: AT4G15090 / FAR1

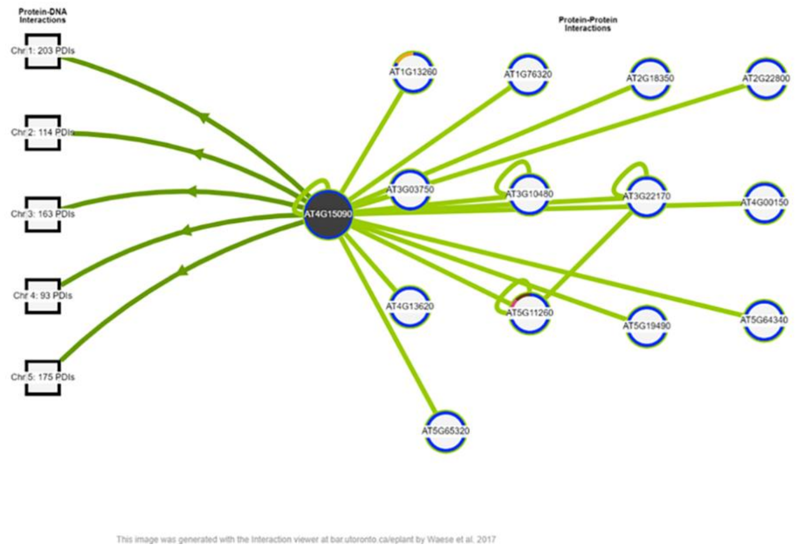

Figure S9. Continued.

(w)

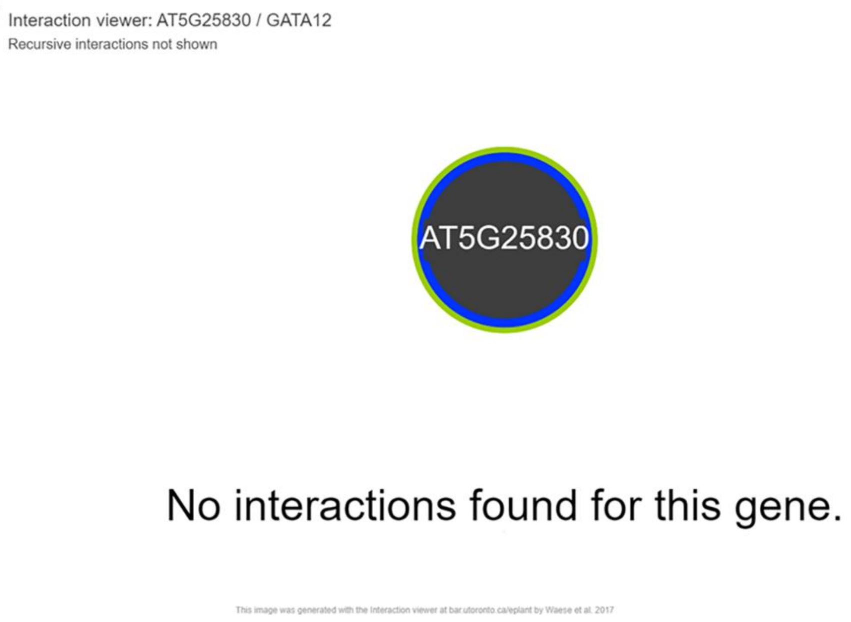

Figure S9. Continued.

Table S1. Suitability of RNA-Sequencing data

Sample	Forward reads		Reverse reads		Mapping rate	Aligned pairs[3)]	Alignment rate
	Input[1)]	Mapped[2)]	Input	Mapped			
Control-whole seedling-1	42,039,414	41,208,192	42,039,414	40,795,349	97.5%	40,607,375	95.2%
Control-whole seedling-2	51,274,070	50,296,108	51,274,070	50,013,253	97.8%	49,770,103	95.9%
NaCl-whole seedling-1	51,728,163	50,850,513	51,728,163	50,516,856	98.0%	50,296,652	95.6%
NaCl-whole seedling-2	40,467,884	39,711,572	40,467,884	39,455,868	97.8%	39,275,051	95.6%
Mannitol-whole seedling-1	37,679,643	36,954,300	37,679,643	36,664,941	97.7%	36,498,403	95.3%
Mannitol-whole seedling-2	42,635,498	41,796,846	42,635,498	41,540,374	97.7%	41,351,177	95.4%
Control-root-1	30,699,480	30,091,441	30,699,480	29,954,951	97.8%	29,818,149	95.3%
Control-root-2	30,259,403	29,502,958	30,259,403	29,435,456	97.4%	29,291,630	94.6%
NaCl-root-1	30,646,933	30,058,038	30,646,933	30,009,796	98.0%	29,853,487	95.3%
NaCl-root-2	30,355,441	27,486,159	30,355,441	27,301,458	90.2%	27,136,922	88.1%
Mannitol-root-1	30,418,358	29,837,792	30,418,358	29,763,872	98.0%	29,623,999	95.9%
Mannitol-root-2	30,633,300	29,664,265	30,633,300	29,579,245	96.7%	29,417,191	94.2%

[1)] Input, trimmed reads; [2)] mapped, mapped reads to reference genome (Arabidopsis genome sequence TAIR 10); [3)] aligned pairs, aligned pairs of forward and reverse reads

Table S3. Expression of the ABA-dependent and -independent genes under osmotic stress conditions in whole seedlings and roots

Gene	Locus ID	NaCl - whole seedling		Mannitol - whole seedling		NaCl - root		Mannitol - root		Pathway
		Fold change	FDR	Fold change	FDR	Fold change	FDR	Fold change	FDR	
RAB18	AT5G66400	38.091	0.013	50.712	0.018	117.821	0.015	216.075	0.042	ABA-dependent pathway
RD29B	AT5G52300	29.849	0.010	38.097	0.024	28.892	0.032	64.291	0.020	
RD22	AT5G25610	4.727	0.011	5.353	0.015	7.872	0.048	9.950	0.050	
RD20	AT2G33380	182.569	0.014	183.186	0.012	7.575	0.047	16.138	0.038	
RD26/ANAC72	AT4G27410	12.753	0.016	15.620	0.016	5.340	0.047	6.738	0.022	
MYB2	AT2G47190	2.880	0.017	3.876	0.031	3.813	0.019	3.118	0.047	
ABF3	AT4G34000	5.092	0.011	5.562	0.022	3.712	0.024	3.469	0.033	
ATAF1/ANAC2	AT1G01720	16.016	0.009	11.089	0.019	2.119	0.049	2.617	0.041	
NCED3	AT3G14440	12.105	0.004	14.173	0.012	1.988	0.039	3.167	0.025	
AREB1/ABF2	AT1G45249	2.586	0.018	2.804	0.022	1.972	0.045	2.520	0.023	
MYC2	AT1G32640	5.997	0.011	6.109	0.014	1.697	0.023	1.357	0.035	
ERD10	AT1G20450	17.483	0.023	19.885	0.014	5.089	0.011	4.745	0.024	ABA-independent pathway
DREB2A	AT5G05410	9.149	0.017	7.572	0.022	1.281	0.016	1.439	0.022	
DREB2B	AT3G11020	2.188	0.042	1.601	0.023	1.674	0.016	1.478	0.047	
COR15A	AT2G42450	250.544	0.023	283.218	0.016	72.846	0.011	92.981	0.037	ABA-dependent and -independent pathway
RD29A	AT5G52310	83.246	0.017	81.067	0.014	35.333	0.021	27.753	0.027	
KIN1	AT5G15960	255.820	0.014	216.036	0.038	33.538	0.032	57.192	0.023	
COR47/RD17	AT1G20440	9.375	0.019	9.817	0.015	3.769	0.048	4.206	0.017	
ERD1	AT5G51070	3.304	0.021	3.017	0.029	1.469	0.014	1.741	0.045	

Table S4. Expression of transcription factor genes upregulated under osmotic stress conditions in roots

Locus ID	Gene Symbol	NaCl - whole seedling		Mannitol - whole seedling		NaCl - root		Mannitol - root		TF family
		Fold change	FDR	Fold change	FDR	Fold change	FDR	Fold change	FDR	
AT1G62975	AT1G62975	9.439	0.013	8.606	0.013	18.839	0.039	14.561	0.004	bHLH
AT3G06490	MYB108	5.270	0.048	4.350	0.020	7.881	0.042	6.032	0.045	MYB
AT5G50360	AITR5	3.169	0.019	4.653	0.020	7.766	0.050	20.205	0.015	DRG
AT4G23550	WRKY29	1.890	0.049	2.100	0.032	5.399	0.050	2.790	0.045	WRKY
AT2G47460	MYB12	2.573	0.034	2.515	0.038	4.801	0.035	4.635	0.033	MYB
AT5G59570	BOA	1.837	0.027	1.941	0.050	3.713	0.047	3.464	0.041	GARP
AT3G24310	MYB71	2.162	0.018	1.729	0.034	3.512	0.015	3.001	0.046	MYB
AT2G47260	WRKY23	2.588	0.018	1.983	0.022	3.344	0.021	3.282	0.027	WRKY
AT1G22640	MYB3	1.219	0.046	1.314	0.030	2.655	0.028	3.643	0.036	MYB
AT1G25560	TEM1	1.203	0.010	1.337	0.045	2.642	0.017	2.092	0.014	AP2/ERF
AT1G51950	IAA18	1.850	0.013	1.894	0.013	2.497	0.018	2.555	0.025	IAA
AT2G45050	GATA2	1.483	0.043	1.572	0.024	2.484	0.020	3.391	0.039	GATA
AT3G07650	COL9	1.453	0.043	1.240	0.037	2.451	0.019	3.012	0.034	B-box zinc finger
AT3G46640	PCL1	1.212	0.031	1.076	0.027	2.358	0.029	2.342	0.039	MYB
AT5G06980	LNK4	1.926	0.037	1.904	0.050	2.341	0.014	3.344	0.047	B-box zinc finger
AT3G23030	IAA2	0.978	0.015	1.446	0.016	2.180	0.025	2.313	0.028	IAA
AT1G19850	MP	1.180	0.016	1.365	0.050	1.985	0.019	2.208	0.042	IAA
AT5G57660	COL5	1.618	0.028	1.684	0.022	1.886	0.026	2.373	0.027	B-box zinc finger
AT4G38620	MYB4	1.163	0.019	1.110	0.039	1.701	0.016	2.051	0.045	MYB
AT2G33310	IAA13	1.027	0.048	0.806	0.028	1.613	0.039	2.024	0.042	IAA
AT5G24800	bZIP9	1.790	0.034	1.430	0.011	1.399	0.021	2.366	0.037	bZIP
AT4G15090	FAR1	1.138	0.028	1.744	0.035	1.383	0.049	2.185	0.011	FRS
AT5G25830	GATA12	1.448	0.016	1.372	0.049	1.372	0.037	2.116	0.046	GATA

Table S5. Expression level of alternative splice variants upregulated under osmotic stress conditions in roots

Gene	Locus ID	Description	Splice variant	NaCl		Mannitol	
				Fold change	FDR	Fold change	FDR
ZIFL1	AT5G13750	zinc induced facilitator-like 1	AT5G13750.1*	1.353	0.049	2.922	0.044
			AT5G13750.2	5.012	0.018	5.110	0.047
			AT5G13750.3	2.252	0.032	2.301	0.035
			AT5G13750.4	3.889	0.039	4.337	0.044
HSFA2	AT2G26150	member of Heat Stress Transcription Factor family	AT2G26150.1*	0.467	0.046	1.713	0.038
			AT2G26150.2	0.943	0.050	1.245	0.050
			AT2G26150.3	0.847	0.050	1.400	0.039
			AT2G26150.4	0.446	0.037	2.474	0.024
UNE6/LUC7A	AT3G03340	LUC7 related protein	AT3G03340.1	1.277	0.049	1.219	0.043
			AT3G03340.2*	1.324	0.043	2.222	0.039
			AT3G03340.3	1.182	0.043	0.881	0.039
RS41	AT5G52040	Encodes an arginine/serine-rich splicing factor	AT5G52040.1	0.925	0.043	0.874	0.37
			AT5G52040.2*	0.806	0.017	0.829	0.049
			AT5G52040.5	1.032	0.050	0.912	0.050
			AT5G52040.3	1.417	0.043	1.000	0.050
			AT5G52040.4	1.723	0.043	2.007	0.046
			AT5G52040.6	0.741	0.049	1.463	0.050
			AT5G52040.7	0.741	0.049	0.940	0.050
SKIP	AT1G77180	Encodes a putative transcriptional factor	AT1G77180.1*	1.273	0.029	1.487	0.048
			AT1G77180.2	2.011	0.014	1.890	0.037
			AT1G77180.3	0.832	0.047	0.926	0.050
AtBBD2	AT1G19660	Encodes a bifunctional nuclease with non-substrate-specific DNase and RNase activity	AT1G19660.1*	0.584	0.046	1.235	0.029
			AT1G19660.2	2.003	0.031	3.145	0.018
			AT1G19660.3	0.861	0.043	1.232	0.045
MYB3	AT1G22640	MYB-type transcription factor	AT1G22640.1*	2.205	0.028	3.581	0.043
			AT1G22640.2	2.383	0.043	1.505	0.041
			AT1G22640.3	2.723	0.045	2.666	0.049
Cor413im2	AT1G29390	Integral membrane protein in the inner envelope of chloroplasts	AT1G29390.1*	2.956	0.029	2.644	0.029
			AT1G29390.2	1.009	0.050	1.739	0.028

(Continued)

Table S5. Continued

Gene	Locus ID	Description	Splice variant	NaCl		Mannitol	
				Fold change	FDR	Fold change	FDR
4CL1	AT1G51680	encodes an isoform of 4-coumarate:CoA ligase	AT1G51680.1*	2.953	0.015	2.791	0.049
			AT1G51680.2	1.013	0.050	1.281	0.047
			AT1G51680.3	1.349	0.046	0.697	0.050
EXPA1	AT1G69530	Member of Alpha-Expansin Gene Family	AT1G69530.1	0.836	0.038	0.819	0.047
			AT1G69530.2	2.416	0.027	2.917	0.038
			AT1G69530.3*	1.239	0.050	1.507	0.037
			AT1G69530.4	1.739	0.031	2.734	0.037
			AT1G69530.5	1.295	0.043	1.084	0.047
AT1G71000	AT1G71000	Chaperone DnaJ-domain superfamily protein	AT1G71000.1*	5.257	0.049	9.776	0.048
			AT1G71000.2	1.582	0.038	2.538	0.044
GRP-3	AT2G05520	Encodes a glycine-rich protein	AT2G05520.1*	4.879	0.039	7.460	0.032
			AT2G05520.2	4.165	0.019	7.855	0.035
			AT2G05520.3	2.780	0.016	6.591	0.027
			AT2G05520.4	3.812	0.043	11.621	0.022
			AT2G05520.5	49.042	0.043	13.809	0.033
			AT2G05520.6	4.005	0.049	7.282	0.042
ADF5	AT2G16700	Encodes actin depolymerizing factor 5	AT2G16700.1*	1.729	0.046	1.882	0.049
			AT2G16700.2	3.763	0.034	2.935	0.044
			AT2G16700.3	1.021	0.050	1.550	0.048
			AT2G16700.4	0.992	0.050	1.346	0.026
AT2G23910	AT2G23910	NAD(P)-binding Rossmann-fold superfamily protein	AT2G23910.1*	5.162	0.025	3.255	0.038
			AT2G23910.2	2.590	0.048	1.628	0.014
			AT2G23910.3	1.926	0.014	1.855	0.046
			AT2G23910.4	0.943	0.046	1.062	0.050
ANNAT4	AT2G38750	Annexins are a family of calcium dependent membrane binding proteins	AT2G38750.1*	12.033	0.032	7.146	0.043
			AT2G38750.2	1.061	0.028	1.030	0.050
MAGL6	AT2G39400	alpha/beta-Hydrolases superfamily protein	AT2G39400.1*	2.925	0.014	3.660	0.050
			AT2G39400.2	1.289	0.016	1.363	0.047
IAA2	AT3G23030	auxin inducible gene expressed in the nucleus	AT3G23030.1	2.171	0.019	2.296	0.043
			AT3G23030.2*	0.869	0.024	1.078	0.037

(Continued)

Table S5. Continued

Gene	Locus ID	Description	Splicing variant	NaCl		Mannitol	
				Fold change	FDR	Fold change	FDR
ICE1	AT3G26744	Encodes a MYC-like bHLH transcriptional activator	AT3G26744.1*	1.377	0.028	2.370	0.041
			AT3G26744.2	2.830	0.029	3.224	0.043
			AT3G26744.4	1.001	0.043	1.000	0.050
			AT3G26744.5	1.603	0.045	1.941	0.027
			AT3G26744.6	1.791	0.036	1.886	0.047
PME34	AT3G49220	Pectin methylesterase	AT3G49220.1	3.181	0.029	3.564	0.036
			AT3G49220.2	1.072	0.050	1.925	0.042
			AT3G49220.3*	1.000	0.047	1.000	0.047
TRM19	AT3G53540	afadin	AT3G53540.1*	3.785	0.013	6.765	0.018
			AT3G53540.2	1.181	0.046	1.327	0.016
AT4G21910	AT4G21910	MATE efflux family protein	AT4G21910.1	4.064	0.037	2.487	0.036
			AT4G21910.2*	1.491	0.036	1.268	0.017
			AT4G21910.3	1.149	0.045	1.128	0.029
			AT4G21910.4	1.153	0.011	1.336	0.37
BCH1/CHY1	AT4G25700	Converts beta-carotene to zeaxanthin via cryptoxanthin.	AT4G25700.1*	2.371	0.026	4.535	0.036
			AT4G25700.2	1.218	0.021	1.477	0.036
			AT4G25700.3	0.796	0.040	1.379	0.046
COR28	AT4G33980	Acts as a key regulator in the COP1-HY5 regulatory hub	AT4G33980.1	2.151	0.024	2.140	0.032
			AT4G33980.2*	1.000	0.043	1.172	0.022
CAD9	AT4G39330	Member of a family of cinnamyl alcohol dehydrogenases	AT4G39330.1*	4.408	0.023	3.501	0.039
			AT4G39330.2	2.150	0.043	1.074	0.047
GORK	AT5G37500	Encodes a guard cell outward potassium channel	AT5G37500.1	2.387	0.015	2.917	0.032
			AT5G37500.2*	1.062	0.050	0.872	0.047
			AT5G37500.3	1.384	0.043	1.644	0.039
			AT5G37500.4	0.927	0.050	1.365	0.036
NUDT2	AT5G47650	Encodes an ADP-ribose pyrophosphatase	AT5G47650.1	2.155	0.020	3.164	0.029
			AT5G47650.2*	2.177	0.029	2.177	0.039
			AT5G47650.3	1.091	0.050	0.818	0.046
			AT5G47650.4	1.350	0.038	1.230	0.039
			AT5G47650.5	1.006	0.043	1.000	0.050

* indicates representative isoform in TAIR

Table S6. List of primers for RT-PCR

Gene	Forward	Reverse
GAPc	5′-GTGTCCCAACCGTTGATGTC-3′	5′-TCCCTTGAGTTTGCCTTCGG-3′
RAB18	5′-TACCAGAACCGTCCAGGAGG-3′	5′-CGTACTCGTCATACTGCTGC-3′
AT1G62975	5′-AGGCAAAGAAGACAAGAAGTTTCA-3′	5′-GTAGTTCACTGCCTGCACGA-3′
AITR5	5′-GAGGGAGAGTATGAGTTAGG-3′	5′-GTTCGTCCAAATGACTCTCC-3′
WRKY29	5′-ATCTGTTGTCGGACGCATGG-3′	5′-TTGCCAAACACCCTTTTGAGC-3′
BOA	5′-TTGGTGGTGGTGACGTGAATG-3′	5′-CAACAACCTCATGAAGTGGGAA-3′
MYB3	5′-AAGCTCCATAGCTTGCTGG-3′	5′-GGCTGAGAAGCTTCCTCTTGA-3′
ANNAT4.1	5′-TCTCAGCTGGGATGGGAATG-3′	5′-GAAACTTTTGCTTGCTTTCC-3′
ANNAT4.2	5′-TTTGTTGTTTATAGGGATGG-3′	5′-GAAACTTTTGCTTGCTTTCC-3′
MAGL6.1	5′-GAAATCAGGTGAGGGAGAAC-3′	5′-GGAATTGATACCTGGTGAAG-3′
MAGL6.2	5′-GAAATCAGGTGAGGGAGAAC-3′	5′-AAAATTAGAACCTGGTGAAG-3′
TRM19.1	5′-CTCACCAAGGAAAGAAACAG-3′	5′-GGAAAATCTTGCTTGTTTTC-3′
TRM19.2	5′-AAGTCATTCATTTTTTCGG-3′	5′-GGAAAATCTTGCTTGTTTTC-3′
CAD9.1	5′-GGAAAGCTCATTGCTTTAGG-3′	5′-CCACGTCACTTCCTCCAACC-3′
CAD9.2	5′- GGAAAGCTCATTGCTTTAGG -3′	5′- TTAGCAAGCTTCCTCCAACC-3′
